# Smoking Knowledge and Behaviors in a Population of Italian Students in Dental Hygiene or Other Health Disciplines

**DOI:** 10.3390/healthcare13101195

**Published:** 2025-05-20

**Authors:** Fabrizio Guerra, Alessia Pardo, Vanessa Di Nasta, Roberta Grassi, Gianna Maria Nardi

**Affiliations:** 1 Department of Oral and Maxillofacial Sciences, “Sapienza” University of Rome, Via Caserta 6, 00161 Rome, Italy; fabrizio.guerra@uniroma1.it (F.G.); giannamaria.nardi@uniroma1.it (G.M.N.); 2 Dentistry and Maxillofacial Surgery Unit, Department of Surgery, Dentistry, Pediatrics and Gynecology (DIPSCOMI), University of Verona, Piazzale L.A, Scuro 10, 37134 Verona, Italy; 3 Independent Researcher, 41100 Modena, Italy; vanessa.dinasta@gmail.com; 4 Department of Biomedical Sciences, University of Sassari, 07100 Sassari, Italy; 5 University of Milano “Vita-Salute San Raffaele”, 20132 Milano, Italy

**Keywords:** smoking, smoke-free products, health students, education

## Abstract

**Background/Objectives**: Smoking remains a major public health concern, and healthcare professionals (HCPs) play a crucial role in smoking cessation efforts. This study aimed to assess the awareness, knowledge, and smoking behaviors of Italian students and graduates in dental hygiene from different regional areas (Group A) and students in health disciplines at a single university (Group B). **Methods**: Two separate surveys were conducted using a specifically designed online questionnaire administered to voluntary participants between February and April 2024. The questionnaire collected data on smoking habits, awareness of smoke-free products, sources of information, and perceptions of health effects. **Results**: A total of 878 questionnaires were completed. While 49.8% of participants had never smoked, 16.3% were regular smokers and 14% were occasional users of either traditional cigarettes or alternative smoke-free products. Awareness of non-combustion products was high, with social circles (74%) and social media (47.9%) being primary sources of information. Users of smoke-free products reported subjective improvements in halitosis, dental discoloration, cough, exercise capacity, and sense of taste. Despite 78% of participants receiving specific training on smoking-related diseases, gaps in knowledge persisted, particularly regarding the toxicological nature of smoking and the role of nicotine in smoking-related diseases. **Conclusions**: These findings highlight the need for continued education and training on smoking cessation and tobacco harm reduction among future HCPs. Strengthening evidence-based knowledge could enhance their ability to manage smoking-related diseases and promote effective cessation strategies.

## 1. Introduction

Tobacco smoking is the greatest threat to health and the second-greatest risk factor for attributable deaths worldwide [1]. According to WHO, there are about 1.3 billion tobacco users around the world and tobacco was responsible, in 2019, for 8.7 million deaths globally [2].

In Italy, a smoking prevalence of 24% is estimated (about 14 million smokers in 2023) with about 96,000 tobacco-attributable deaths per year [3].

Cigarette smoking represents the single most widely preventable cause of cardiovascular disease and tumors, and cessation is the most effective intervention to reduce the risk [4,5,6,7].

As for oral health, smoking is a risk factor for the development of periodontal disease, and for its remission [8,9,10]. Smoking also plays a key role in some diseases of the oral mucosa: primarily squamous cell carcinoma, which affected over 300,000 people worldwide in 2012 and was diagnosed in 371,000 people in 2019 in Italy [11,12,13]. Leukoplakia, chronic hyperplastic candidiasis, nicotinic stomatitis, and melanosis are other smoking-related pathologies [14,15,16,17,18,19,20].

Currently, there are specific guidelines for the smoking cessation process, which establish the following key components: (1) educational aspects, information intervention, learning; (2) behavioral support; (3) Nicotine Replacement Therapy—NRT—and pharmacological therapy [21].

Alongside cessation, for those adult smokers who decide to keep on smoking, the so-called tobacco harm reduction (THR) principle has been considered. Harm reduction concept refers to interventions aimed at reducing the negative effects of health behaviors without necessarily extinguishing such problematic behaviors completely or permanently [22]. As the harmful effects of cigarette smoking on health are not mainly induced by nicotine, but by the toxic substances produced during the combustion of tobacco [23,24] and defined by the US Food and Drug Administration (FDA) as “Harmful or Potentially Harmful Constituents” (HPHCs) [25]. The THR objective is to completely transition adult cigarette smokers who decide to continue smoking, to consuming nicotine in ways other than burning tobacco, to significantly reduce their exposure to combustion products and therefore, potentially, the resulting harm [26,27,28,29,30].

Both dentists and oral hygienists are in the unique position of regularly seeing patients who smoke. Helping and encouraging them in their battle against smoking is an important way to contribute to improving patients’ health [31]. The opportunity to frequently discuss correct lifestyles leads them to play an important prevention role: identifying smokers, offering minimal advice support, documenting their clinical history, and providing supporting information materials, as an integral part of clinical practice.

However, healthcare providers (HCPs) are experiencing several difficulties in properly addressing a real global health emergency. Physicians, when examining patients with tobacco-related diseases for the first time, are often limiting their intervention in providing a generic recommendation to quit smoking. Taking care of patients with appropriate counseling or a more structured intervention focused on cessation is seldom adopted.

A review of the results of some surveys on HCPs from different health disciplines, conducted some years ago in Italy, showed that the sensitivity of both General Practitioners and Specialists to smoking issues is fair, even if there is a discrepancy between individual HCPs and Scientific Societies in the level of interest in risk-reduced smoking devices. Even though the smoking history is collected by most physicians—together with providing advice to quit—the adherence to smoking addiction guidelines was still unsatisfactory [32].

A further survey conducted by Censis in 2022, on a sample of Italian smokers and physicians, showed that the physician’s role in addressing patients’ smoking behaviors is extremely limited: own General Practitioner is not considered an actual reference by most of the interviewed smokers and the latter are receiving information on smoking and alternative products to cigarettes mainly by web, word of mouth, and commercial channels. Physicians themselves admitted to obtaining self-managed information about smoking, cessation, and alternative products and to seldom talking to their patients about smoking issues in their daily clinical practice [33,34].

Therefore, to further explore the smoking behaviors and the level of awareness/knowledge about smoking issues in Italian HCPs, we conducted two separate surveys. One was carried out in both students and graduates in oral hygiene from different Italian regional areas and the other one in students of different HCP disciplines from the University of Modena.

Our main aim was to identify potential room for improvement in the approach and management to smoking issues in young HCPs, who could play a future key role in the fight against smoking and in helping smokers to optimize their quitting efforts or reduce the risk of tobacco-induced harm as much as possible, if they decide not to quit.

## 2. Materials and Methods

### 2.1. Study Design

This study examined the awareness, knowledge, and smoking behaviors of Italian healthcare students and graduates. This evaluation was conducted through a cross-sectional survey, adhering to the STROBE checklist for observational studies [35]. Two distinct surveys, designated as Surveys A and B, were employed to gather data from different target populations.

### 2.2. Study Setting and Population

To conduct the survey, a specific questionnaire focusing on smoking behaviors and knowledge of smoking-related issues was developed (see also Supplementary Materials) and made available online for participants to voluntarily complete. These participants were divided into two groups:

Group (A)—students and graduates in oral hygiene from different regional areas in Italy.

Group (B)—students in different health disciplines (Medicine and Surgery; Dentistry; Nursing; Obstetrics; Physiotherapy; Psychology; Dietetics; Speech Therapy; Occupational Therapy; Techniques of Radiology; Rehabilitation; Pathophysiology; Laboratory; and Healthcare) attending the University of Modena and Reggio Emilia (UNIMORE).

The survey for Group A was conducted from 15 February to 16 April 2024, while the survey for Group B took place between 18 February and 11 April 2024. The study protocol received approval from the Institutional Review Board of the Department of Oral and Maxillofacial Sciences at Sapienza University of Rome (Protocol n.1418-21/09/2022).

Before filling in the survey, each participant was requested to provide informed consent on the data collection and use of the results for research purposes.

Answers to each question were entered in a cumulative spread sheet and the different rates for each type of answer were then automatically calculated.

The questionnaire has been developed based on previous ones adopted by similar surveys conducted by Scientific Societies in Italy [32]. Methods for developing and validating the questionnaire are described in detail elsewhere [36]. The questionnaire was reviewed by experts (A.P., G.M.N.) to ensure its relevance and suitability for the target population. Before being implemented, it was piloted with a small group of participants (10 dental hygienist students) to evaluate its clarity, comprehension, and ease of use. Feedback from pilot participants was systematically collected and analyzed to identify areas for improvement and refinement. The final version of the questionnaire integrated feedback from expert reviews, pilot testing, and reliability analysis to enhance its validity and reliability in evaluating smoking behaviors and knowledge among the target population. It was designed for administration through various modes, including online platforms and in-person interviews, to maximize accessibility and response rates. Clear instructions and definitions were provided to respondents to minimize ambiguity and ensure consistent interpretation of questions across participants.

### 2.3. Participants

Eligibility Criteria for Inclusion: Participants must either be (a) students or graduates in oral hygiene from various regional areas in Italy (for Group A), or (b) students in specified health disciplines (for Group B). Participants are required to provide informed consent for data collection and the use of results for research purposes, and they must complete the online questionnaire within the designated survey periods for Group A and B. Exclusion Criteria: Individuals who are not students or graduates in oral hygiene from Italian regional areas (for Group A) or not students in specified health disciplines (for Group B) are excluded. Additionally, participants who do not provide informed consent for data collection and research use, those who complete questionnaires outside the specified survey periods for each survey, incomplete questionnaires or those with missing responses, submissions not made through the designated online platform, individuals unable to access or complete the online questionnaire independently, responses from participants who do not voluntarily fill in the questionnaire on smoking behaviors and knowledge, duplicate submissions from the same participant, questionnaires completed by individuals who do not meet the age requirements (if any specified in the study protocol), and responses from participants who withdraw consent during or after the survey completion are also excluded.

### 2.4. Measurement

For the measures in this study, the following variables were collected: 1. Smoking habits: Categorized into regular smokers, occasional smokers, ex-smokers, and non-smokers. 2. Awareness of smoke-free products: Measured using awareness levels (aware, somewhat aware, and not aware). 3. Perceptions of health effects: Subjective improvements reported by users of smoke-free products in areas such as halitosis, dental discoloration, cough, exercise capacity, and sense of taste. 4. Knowledge of smoking-related diseases: Assessed through questions about specific training received and understanding of the toxicological nature of smoking and nicotine’s role in smoking-related diseases. We used a scale from 1 (low risk) to 10 (high risk). 5. The importance of the role of nicotine in the development of disease: assessed with a scale of extremely important, very important, important, not important, and no contribution. The total sample size (N) was reported for each variable, with information on missing data included where applicable. Data presentation considered stratification by relevant demographic factors such as age groups and sex. Categories were properly labeled, and percentages were clearly presented to facilitate easy interpretation of descriptive statistics.

### 2.5. Statistical Methods

The analysis focused on summarizing and presenting the distribution of responses across various categories. We performed descriptive statistics for categorical variables: 1. Smoking habits: The frequencies and percentages for each category (e.g., daily smokers, occasional smokers, former smokers, and non-smokers) were calculated. 2. Awareness of smoke-free products: We computed the frequencies and percentages for different awareness levels (e.g., aware, somewhat aware, and not aware). The findings are illustrated using bar charts or tables. We reported the total sample size (N) for each variable. We included information on missing data, if applicable, and considered presenting data stratified by relevant demographic factors (e.g., age groups and sex).

## 3. Results

A total of 1200 students from various universities were invited to participate in the study. Of these, 473 questionnaires were completed by students specializing in dental hygiene, while 405 were completed by students from the medical/health disciplines, including physicians, nurses, dentists, obstetricians, physical therapists, psychologists, rehabilitation technicians, speech therapists, radiology technicians, pathophysiology technicians, dieticians, occupational therapists, laboratory technicians, and healthcare assistants (Table 1).

Table 1 presents data on demographics and training, smoking behavior, knowledge and information sources, and product use among users, as well as occasional smoking data and the types of e-cigarettes used. Table 2 provides information on smoking cessation methods, reasons for smoking combusted products, reasons for using smoke-free products, and health improvements observed after switching.

### 3.1. Group A

A sample of 473 students/graduates filled in the online questionnaire.

The age range of the participants was 19–78 years with a median age of 29 years (81% female, 18.8% male and 0.2% other).

In 59.2% of cases, they were graduates (no. = 280), while the students (no. = 193) belonged to the first (10.8%), second (17.2%), and third (12.7%) year of their degree.

Among the institution regions in which the questionnaire was administered (Piedmont, Liguria, Lombardy, Trentino Alto-Adige, Veneto, Friuli-Venezia Giulia, Emilia-Romagna, Tuscany, Sardinia, Lazio, Abruzzo, Campania, and Puglia), the most represented were Emilia-Romagna (22.7%), Veneto (14.4%), Lombardy (12.2%), and Piedmont (11.5%); the least represented was Sardinia (0.7%).

Specific training on smoking, smoking-related diseases, smoking cessation methods, and alternative products was reported by 59%, 78%, 38.7%, and 36% of respondents, respectively.

**Figure 1:**
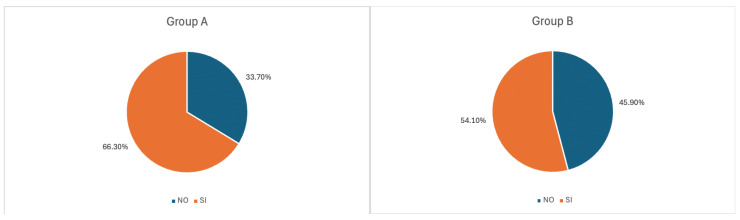
Rates of tobacco product use among regular users in Group A and B.

Most respondents had never smoked (49.8%) while smokers or users of non-combustion products (heated tobacco products—HTPs, electronic cigarettes—E-cigs) were regular in 16.3% of cases and occasional in 14%. Also, 19.9% of respondents were ex-smokers (Figure 1).

Ex-smokers have been smokers for a variable amount of time, with a distribution of the different percentages over different durations: the highest percentage was reached in ex-smokers who previously smoked for 2 years (9.4%). In most cases, they were ex-cigarette smokers (82.9%) who smoked mainly 4–9 cigarettes a day.

In many cases (91.3%), the respondents had heard of combustion-free products. E-cigs were known by almost all respondents (99.8%), while HTPs were known in 84.2% of cases.

Respondents who knew about alternative combustion-free products learned about them (multiple answers possible) thanks to friends (74.3%), social media/web (47.9%), scientific conferences/workshops (16.5%), family members (15.6%), and newspaper articles (15.4%).

Occasional smokers had been such smokers for 1 year (8.3%), 3 years (11.1%), 5 years (12.5%), or 10 years (11.1%); the rates of other occasional smokers were more dispersed between the different durations.

Regular smokers did so for a period varying from 1 to >40 years, with a somewhat dispersed distribution in rates between the different durations; the highest percentage recorded was 3 and 4 years, each consisting of 10% of the respondents.

**Figure 2:**
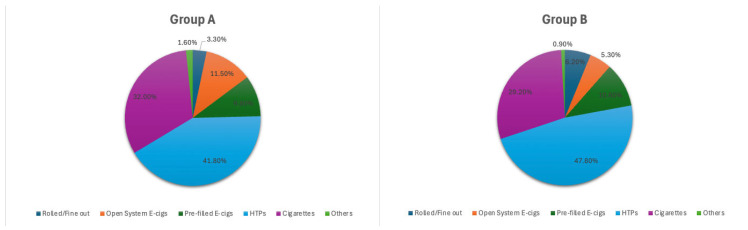
Rate of respondents showing improvement in health status after switching from cigarettes to smoke-free products, and kind of reported improvements.

Among smokers and/or users of smoke-free products, the majority used HTPs (41.8%), followed by cigarettes (35.3%) and E-cigs (21.3%) (Figure 2). The majority of cigarette smokers smoked 1–3 or 4–9 a day, while the use of E-cigs with pre-filled sticks appeared to be more or less equally diversified (1–3, 4–9, 10–15, 16–20, >20 per day); most open-system E-cig users used between 1–3 and 4–9 per day; for HTPs, the main use was 1–3 and 4–9 sticks per day, followed closely by 16–20 sticks per day.

Occasional smokers mainly used cigarettes (45.8%) while E-cigs were used by 32.6% and HTPs by 16.9%.

Most regular cigarette smokers smoked <5 cigarettes per day. HTPs users, on the other hand, showed a uniform quantitative distribution in the various amounts considered (<5, 6–10, 10–15, 16–20 sticks/day).

Most occasional users of tobacco products did so <5 days/month (66.7%). This was followed by 6–10 days/month (15.2%), 16–20 days/month (12.1%), and 10–15 days/month (6.1%).

As for E-cigs, the rate of users of closed and open systems were similar (39.3% and 41%, respectively), while disposable non-rechargeable E-cigs were used by 19.7% of E-cig users.

Regarding cessation, most did it spontaneously (89.2%), with just 1% of cases going to a no-smoke center.

Smokers of combusted products started them for the following reasons: “reduce stress” (37.2%); “sense of integration/belonging” (36.2%); “personal pleasure from tobacco” (26.6%); or “emulation” (18.1%). Only 7.4% reported having done it to “increase their concentration skills”.

Instead, for smoke-free products, the main reasons for use were “perception of lower health risk” (59.1%); “possibility of using them even in closed environments” (33%); “stress reduction” (26.1%); “personal pleasure from tobacco” (20.9%); and “sense of integration/belonging” (12.2%). The percentage of those who chose them for “emulation” was limited (7%) and even less when they believed there is an “absence of harm to health” (2.6%).

For those who switched from cigarettes to smoke-free products, this occurred in many cases after 2 years (23.25%), 3 years (23.2%), and 4 years (10.1%). Most respondents who switched from cigarettes to alternative products showed subjective improvements in their health status (66.3%).

**Figure 3:**
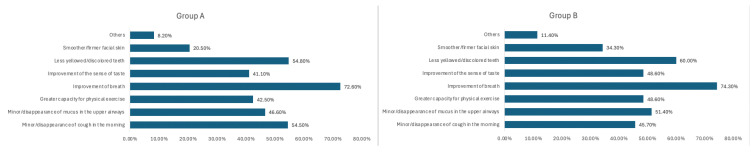
Self-perceived improvements in health status in Group A and B.

The self-perceived improvements noted after switching to these products were (more than one could be selected): reduction/disappearance of halitosis (72.6%); less yellowed/discolored teeth and reduced/disappeared cough (54.8% each); lower production/disappearance of mucus (46.6%); improved physical exercise skills (42.5%); improvement of the sense of taste (41.1%); smoother/firmer facial skin (20.5%); and other (8.2%) (Figure 3).

The perception of health risk relating to cigarettes was mostly placed at the maximum value, while it was equally distributed in the case of non-combusted products and in any case significantly lower than that of cigarettes.

Among the smoke components, tar and carbon monoxide were particularly perceived as risky, followed by inhaled smoke and nicotine.

Nicotine was perceived as having an important (17.1%), very important (28.4%), or extremely important (52%) role in smoking harm. The same perception was maintained for nicotine as the presumed culprit in the development of lung tumors: important (18%); very important (22.9%), or extremely important (52.2%). The same applied to the role of nicotine in the development of tumors in other organs: important (30.3%), very important (29.8%), or extremely important (32.3%). And again, the same negative perception of nicotine was recorded for the role it has in the development of COPD: important (22.5%), very important (23.7%), or extremely important (48%).

Most participants considered the training of dental hygienists on problems related to smoking/alternative products to be useful or fundamental (98.2%).

### 3.2. Group B

A sample of 405 students of different health disciplines at the University of Modena and Reggio Emilia filled in the online questionnaire.

The health professions involved included physicians (*n* = 126; 31%), nurses (*n* = 73; 18%), dentists (*n* = 32; 8%), obstetricians (*n* = 28; 7%), physiotherapists (*n* = 25; 6%), psychologists (*n* = 24; 6%), rehabilitation technicians (*n* = 20; 5%), speech therapists (*n* = 19; 4.6%), radiology technicians (*n* = 19; 4.6%), pathophysiology technicians (*n* = 11; 2.7%), dieticians (*n* = 10; 2.4%), occupational therapists (*n* = 9; 2.2%), laboratory technicians (*n* = 7; 1.7%), and healthcare assistants (*n* = 2; 0.4%).

The age range of participants was 18–41 years, with a median age of 21 years (74.1% female, 25.4% male, and 0.5% other).

Specific training on smoking, smoking-related diseases, smoking cessation methods, and alternative products was reported by 48.4%, 67.6%, 23%, and 22.8% of the respondents, respectively.

Most respondents had never smoked (45.90%) (Figure 1), while smokers or users of non-combustion products (HTPs, E-cigs) constituted 21.5% of cases (Figure 1) and occasional users in 17.1%. Finally, 5.7% of respondents were ex-smokers.

Ex-smokers have been smokers for a variable amount of time, with distribution of the different rates over different durations: the highest rate was reached in ex-smokers for 1 year and 5 years (14.8% in each case). In many cases, they were ex-cigarette smokers (55.2%) who smoked mainly 1–3 a day.

In most cases (88.1%) the respondents had heard of combustion-free products. E-cigs were known by almost all respondents (99.7%) while HTPs were known in 79.1% of cases.

Respondents who knew about alternative combustion-free products learned about them (multiple answers possible) thanks to friends (72%), family members (2.7%), or social media/web (23.1%).

The percentages of those who learned about it thanks to scientific conferences/workshops and newspaper articles were quite low.

Occasional smokers had been smokers mainly in the past 2 years (15.9%), 5 years (11.6%), and 3 years (10.1%), as well as 7.2% for 1 year; the percentages of other occasional smokers were more dispersed between the different durations.

Regular smokers did so for a period varying from 1 to 20 years, with a somewhat dispersed rate distribution between the different durations: smoking since 3 and 4 years ago had relatively higher rates of respondents (each 13.8%).

Among smokers and/or users of smoke-free products, the majority used HTPs (47.8%), followed by cigarettes (35.4%) and E-cigs (10.6%) (Figure 2).

Many cigarette smokers smoked 4–9 cigarettes a day, as did users of HTP sticks, while the use of E-cigs with an open system appeared to be equally diversified (1–3 mL, 4–9 mL, and 10–15 mL per day).

Occasional smokers mainly used cigarettes (60.3%), while E-cigs were used by 20.6% and HTPs by 16.4%.

Most cigarette smokers smoked <10 cigarettes per day. HTPs users, on the other hand, mostly used 4–15 sticks/day.

Most occasional users of tobacco products do so <5 days/month.

Regarding E-cigs, the rate of users of closed and open systems were similar (36.2% and 46%, respectively), while disposable non-rechargeable E-cigs were used by 17.2%.

In the case of quitting, as many as 89.3% did so spontaneously and none attended no-smoke centers.

Smokers of combusted products started them for the following reasons: “reduce stress” (60.9%); “personal pleasure from tobacco” (39.1%); “sense of integration/belonging” (32.6%); and “emulation” (25%). Only 13% reported having done it to “increase their concentration skills”.

For alternative products, the main reasons for use were “perception of lower health risk” (55.8%); “stress reduction” (44.2%); “possibility of using them even in closed environments” (40.4%); “personal pleasure from tobacco” (23.1%); “sense of integration/belonging” (18.3%); and “emulation” (16.3%). The percentage of respondents who use them because they believed there was an “absence of health harm” was very low (3.8%).

For those who switched from cigarettes to smoke-free products, this occurred in most cases between 3 and 4 years (15.1% in each case), 2 years (13.2%), and 1 and 5 years (5.7% in each case).

Respondents who switched from cigarettes to alternative products reported self-perceived improvements in their health status in 45.9% of cases.

The reported changes noted after switching to these products were as follows (more than one could be selected): reduction/disappearance of halitosis (74.3%); less yellowed/discolored teeth (60%); lower production/disappearance of mucus (51.4%); improved sense of taste and improved exercise capabilities (48.6% in each case); improvement/disappearance of cough (45.7%); smoother/firmer facial skin (34.3%); and other (11.4%) (Figure 3).

The perception of health risk related to cigarettes was in many cases positioned at the maximum value, while it was equally distributed in the case of non-combusted alternative products (always lower than that of cigarettes).

Among the components of smoke, tar and carbon monoxide were particularly perceived as risky, followed by inhaled smoke and nicotine.

Nicotine was perceived as having an important (27.4%), very important (32.6%), or extremely important (33.6%) role in the development of smoking-related diseases. The same perception is held for nicotine as the presumed culprit in the development of lung tumors: important (29.5%); very important (25.9%), or extremely important (28.2%).

The same applied to the role of nicotine in the development of tumors in other organs: important (26.9%), very important (39.4%), or extremely important (15%). And again, the same negative perception of nicotine was recorded for the role it has in the development of COPD: important (27.7%), very important (26.4%), or extremely important (29.8%).

Most respondents considered the training of various healthcare professions on problems related to smoking/alternative products to be useful or fundamental (95.6%).

## 4. Discussion

There is considerable room for improvement regarding smoking in terms of specific training which involves all levels of education, both at the level of undergraduate and post-graduate schools’ courses. This often leads to a lack of involvement in smoking cessation processes, believing that quitting smoking is a private matter for the patient, linked more to a bad habit rather than to an addiction.

Our surveys integrated previous results from several investigations conducted so far in Italy by Scientific Societies, the results of which have been made public, on the attitude of General Practitioners and Specialists towards problems relating to smoking [32].

Despite our surveys being conducted in different samples (one in a mixed population of students/graduates from different Italian geographical regions and the other on students from a single university), there are some common features which are worth discussing and speculating about.

Overall, the survey sample was mostly formed by students (598 out of 878: 68%) either graduating in oral hygiene or other health disciplines, hence providing a sort of “snapshot” of young health-educated people in terms of their smoking behaviors and awareness/education on smoking issues.

It is reassuring that in such a population, the rate of non-smokers and ex-smokers represents most of the participants. On the other hand, the rate of cigarette smokers was still quite high: 44.3% and 38.6% in group A and B, respectively.

These results align with those of the study by Nakano et al. [37], which reported a smoking prevalence of 20.3%, including both current and occasional smokers. This contrasts with the study by Jacquelyn et al. [38], which assessed anti-tobacco-use behaviors and attitudes among licensed dental hygienists in five states and the District of Columbia, finding that only 6.9% of the sampled hygienists were current smokers. However, in both surveys, among those who were using a tobacco product, the rate of cigarette smokers (35.3% and 35.4% in survey A and B, respectively) was limited in comparison to the use of smoke-free products: 63.1% and 63.7%, respectively, in survey A and B. These findings could suggest that such this population received a specific education on smoking-related diseases (78% in survey A and 67.6% in survey B), in line with data found in the literature [39]. It is not surprising that many respondents chose the use of smoke-free product on the basis of perception of lower health risk (59.1% and 55.8% in survey A and B, respectively), which is in line with the THR concept of considering the maximum risk level for cigarette smoking in adults, and assigning cessation with zero risk and smoke-free products with an intermediate risk [26,27,28,29,30,40]. Of note, even if the age range was different between the two surveys (19–78 years, median 29 years in survey A; and 18–41, median 21 years in survey B), the perception of health risk, which oriented the choice of smoke-free products, did not differ much (about 3 percentage points).

Certainly, it would have been better recording an even wider rate of non-smokers/ex-smokers, but that could be a consequence of the relatively low specific education received by participants on smoking cessation (38.7% and 23%, respectively, in survey A and B), which was affecting both their personal behaviors and could limit the future effectiveness in helping patients to quit. Apparently, dental hygienists (both graduates and students) were the ones better educated on smoking cessation, which can be the result of the training efforts carried out so far by various scientific dental hygiene societies in Italy. Walsh et al. studied the impact of continuing education on the mechanisms and models of smoking cessation. Their data revealed a significant improvement in knowledge and behavior among 1463 dental hygienists six months after completing the course, compared to baseline values [39].

There are also two other emerging and interesting findings in the survey’s results.

Firstly, participants who used smoke-free products in both surveys reported subjective improvements in their health status after switching from cigarettes, particularly as to halitosis, dental discoloration, cough and production of mucus, sense of taste, and exercise capability, which is in line with previous findings from several clinical studies [40,41,42].

Secondly, even if most of the participants in both surveys declared to have received specific training on smoking-related diseases and considered tar and carbon monoxide the riskiest components of combusted smoking, the negative misperception of nicotine’s role is quite puzzling.

Nicotine, although addictive and not without risks, is not the primary cause of smoking-related diseases. Cigarette smoking contains thousands of chemicals (HPHCs). It is this mixture of chemicals—not nicotine—that causes serious illness and death in smokers, including lung and other types of cancer, COPD, etc. [43,44].

Even if it has been reported that 4 out of 10 smokers and ex-smokers incorrectly think that nicotine causes most smoking-related cancers, it is surprising that such a misperception still is present among a group of supposedly well-health-educated people.

However, it is reassuring in some way that most respondents, in both surveys, considered the training of various healthcare professions on problems related to smoking/alternative products to be useful or fundamental, and therefore an area which is worth continuing to invest in and utilize for the benefit of both HCPs and their patients.

The potential role of alternatives to cigarette smoking in terms of oral health benefit is still debated and several systematic (with or without metanalysis) and non-systematic reviews have been published so far [45,46,47]. Whilst the current evidence on the absolute risk of smoke-free products shows that they cannot be considered risk-free (and therefore have a negative oral health impact), the comparison in terms of their relative risk versus that of cigarette smoking is, on the contrary, quite favorable [48,49,50,51,52,53].

Of course, there are many limitations to our surveys. The first one is due to the nature of self-reported surveys, whose inherent bias can limit their direct application to health policies [54], which is of course to be taken into account even if it is unavoidable. The second is mainly due to the voluntary participation of two samples of students/graduates that could not be fully representative of such population behaviors/attitudes/knowledge at a national level.

The main strengths and limitations are as follows. Strengths: The large sample size of 878 participants offers a comprehensive overview of smoking behaviors and awareness among young, health-educated individuals. These include both students and graduates from various health disciplines. The study also compares data from two separate surveys to reveal consistent patterns. The evaluation covers both smoking behaviors and knowledge/education on smoking-related issues.

The implications for practice highlight the need for enhanced education on smoking cessation techniques across all health disciplines. It is crucial to address misconceptions about nicotine, particularly among health professionals. Dental hygienists, in particular, have the potential to play a more significant role in smoking cessation efforts, and there is an opportunity to integrate harm reduction concepts into health education curricula.

## 5. Conclusions

Although our results were somewhat better than those from previous surveys conducted on physicians, there remains a notable lack of recognition of evidence, particularly of a toxicological nature, and only partial information on alternatives to combusted smoking among students and graduates in dental hygiene and other health professions. However, the former group appeared to be better trained in smoking-related diseases and risk issues, with a 10% difference between group A and B. Overall, our findings underscore the need to reinvigorate the fight against smoking by implementing strategies focused on prevention and cessation, and supporting risk reduction actions for adult smokers who have not quit. It would be beneficial to formally integrate aspects related to smoking-related pathologies, cessation policies, and risk containment into the degree courses of various scientific disciplines, as well as to address the new alternatives to cigarettes that are becoming increasingly popular among both health workers and their patients. To achieve this goal, and based on our findings, we suggest the following priorities: (A) Enhance academic training and information for students and healthcare providers regarding lifestyle changes focused on smoking issues, cessation, and risk reduction/minimization. (B) Openly discuss and share available evidence on smoking harm within the scientific community and with patients, monitoring its effects and fostering a strong and effective patient–healthcare worker relationship.

## Figures and Tables

**Table 1:** General characteristics and outcomes of survey A and B.

Demographics and Training	Group A (%)	Group B (%)
Female	81.0	74.1
Male	18.8	25.4
Other gender	0.2	0.5
Training: smoking	59.0	48.4
Training: smoking diseases	78.0	67.6
Training: cessation methods	38.7	23.0
Training: alternative products	36.0	22.8
Smoking Behavior		
Never smoked	49.8	45.9
Regular smokers	16.3	21.5
Occasional smokers	14.0	17.1
Ex-smokers	19.9	5.7
Knowledge and Information Sources		
Heard of E-cigs	99.8	99.7
Heard of HTPs	84.2	79.1
Info source: friends	74.3	72.0
Info source: social/web	47.9	23.1
Info source: family	15.6	2.7
Info source: conferences	16.5	-
Info source: newspapers	15.4	-
Product Use Among User		
HTPs	41.8	47.8
Cigarettes	35.3	35.4
E-cigs	21.3	10.6
Occasional Smoking Data		
Cigarettes	45.8	60.3
E-cigs	32.6	20.6
HTPs	16.9	16.4
Data on E-cig Type Used		
Closed system	39.3	36.2
Open system	41.0	46.0
Disposable	19.7	17.2

Abbreviations: HTPs (heated tobacco products), HCPs (healthcare professionals), and E-cigs (electronic cigarettes).

**Table 2:** Data on methods of smoking cessation and other types of smoking product.

Method of Smoking Cessation	Group A (%)	Group B (%)
Spontaneous cessation	89.2	89.3
No-smoke center	1	0.0
Reason for smoking combusted product		
Stress reduction	37.2	60.9
Sense of belonging	36.2	32.6
Pleasure from tobacco	26.6	39.1
Emulation	18.1	25.0
Increase concentration	7.4	13.0
Reason for using smoke-free product		
Lower health risk perception	59.1	55.8
Use in closed environments	33.0	40.4
Stress reduction	26.1	44.2
Pleasure from tobacco	20.9	23.1
Sense of belonging	12.2	18.3
Emulation	7.0	16.3
Perceived absence of harm	2.6	3.8
Health improvement after switching		
Halitosis reduction	72.6	74.3
Less yellowed teeth	54.8	60.0
Less mucus	46.6	51.4
Better exercise capacity	42.5	48.6
Improved taste	41.1	48.6
Less coughing	54.8	45.7
Better skin	20.5	34.3
Other	8.2	11.4
Perception of nicotine’s role in disease		
General harm	52.0	33.6
Lung tumors	52.2	28.2
Other tumors	32.3	15.0
COPD	48.0	29.8
Perceived importance of training		
Considered useful or fundamental	98.2	95.2

Abbreviation: COPD (chronic obstructive pulmonary disease).

## Data Availability

Data are available upon reasonable request to the corresponding authors.

## Supplementary Materials

The following supporting information can be downloaded at: https://www.mdpi.com/article/10.3390/healthcare13101195/s1.
