# Supplementary material for: Smoking Knowledge and Behaviors in a Population of Italian Students in Dental Hygiene or Other Health Disciplines
Source: Healthcare (Basel). 2025 May 20;13(10):1195. doi: 10.3390/healthcare13101195 (PMC12110899; doi:10.3390/healthcare13101195)
Supplement: Supplementary file 1 [file healthcare-13-01195-s001.zip › healthcare-3526231-supplementary.pdf]

Survey A

TRACKLING CIGARETTE SMOKING: LEVEL OF AWARENESS AND BEHAVIOR OF STUDENTS IN HEALTH PROFESSIONS: UNIVERSITY OF MODENA AND REGGIO EMILIA

Participant Characteristics

1. Age
2. Gender
3. Year of Dental Hygiene Degree Course
4. Indicate the region in which you study or have studied
5. Indicate the region in which you practice the profession
6. Have you ever had specific training in smoking? - Smoking-related diseases Methods for quitting smoking - alternative products?
7. Are you a smoker?
8. If you smoke regularly, how long have you smoked? Express in months/number of years
- No, I have never smoked
- Yes, regularly
- Yes, occasionally
- No, I am an ex-smoker
9. What do you smoke?
- Traditional cigarettes
- Cigars
- Cigarillo
- Pipe
- Pre-filled e-cigarette sticks
- Refillable e-cigarette with liquids
- Heated tobacco
- Rolled or fine cut
10. How often?
-1/3 per dayszd
- 4/9 per day
- 10/15 per day
-16/ 20 per day
- >20 per day
11.If you smoke occasionally, how long have you smoked? Express in months or years
12. If you are an occasional smoker, what do you smoke?
- Traditional cigarettes
-Cigars
- Cigarillo
- Pipe
- Electronic cigarette pre-filled sticks
- Electronic cigarette with refillable liquids- Heated tobacco- Rolled or fine cut
-1/3 per day, - 4/9 per day, - 10/15 per day-16/ 20 per day- >20 per day

13. How often? Indicate the number of days/month (if you smoke >20 days per month you are not an occasional smoker)
- Traditional cigarettes
- Cigars

- Cigarillo
- Pipe
- Electronic cigarette pre-filled sticks
- Electronic cigarette with refillable liquids
- Heated tobacco
- Rolled or fine cut

-1/3 per day, - 4/9 per day, - 10/15 per day-16/ 20 per day- >20 per day

14. I am an ex-smoker. How long? Express in months-years

15.What did you smoke?

- Traditional cigarettes
- Cigars
- Cigarillo
- Pipe
- Electronic cigarette pre-filled sticks
- Electronic cigarette with refillable liquids
- Heated tobacco
- Rolled or fine cut

16. How often?

-1/3 per day
- 4/9 per day
- 10/15 per day
-16/ 20 per day
- >20 per day

17. If you are a smoker, how would you quit smoking (multiple answers possible)?

- Anti-smoking Center
- Spontaneously
- Nicotine Replacement Therapy (Nicotine gum, patches, spray)
- Pharmacologically (bupropion, varenicline, cytisine)
- Anti-smoking counseling
- Alternative therapies
- Other

Approach to smoking/use of alternative products

18. If you were a smoker of combustible products, what was the main motivation that led you to start?

- Emulation
-Personal pleasure of tobacco
-Sense of integration/belonging to a group
-Personal discomfort
-Reduction of stress
-Increased ability to concentrate
-Other

19. If you are a user of alternative products without combustion (electronic cigarette and heated tobacco), what was the main motivation that led you to start/switch to the latter (multiple answers possible)?

- Emulation
-Personal pleasure of tobacco
-Sense of integration/belonging to a group
-Personal discomfort
-Insecurity

-Reduction of stress
-Increased ability to concentrate
- Perception of lower health risk compared to traditional cigarettes
-Absence of health dangers
-Possibility of using them indoors
-Other

20. If you have switched from traditional cigarettes to smokeless products:

How long ago? Express in months or years. (e.g. 1 year)

21. Since the switch to today have you noticed any changes in your health/aesthetics?
- yes
-no

22. If yes, please tick one or more of the following boxes:
- Less/disappearance of cough in the morning
- Less/disappearance of mucus in the upper airways
- Greater ability to exercise
- Improved breath
- Improved sense of taste
- Less yellow/discolored teeth
- Smoother or firmer facial skin
- Other
23. If you use e-cigarettes, what type?
- open system (refillable tank)
- closed system (pre-filled cartridges)
- disposable, non-refillable

Knowledge
Using a scale from 1 (low risk) to 10 (high risk) and based on the information you have, indicate what, in your opinion, is the score associated with the health risk of the following products and components for smokers:

24. Health risk score for:
- Traditional cigarettes
- Electronic cigarettes (e-cigs)
-Nicotine replacement therapy
-Heated tobacco products
25. Health risk scores for smoking components
- Nicotine
- Inhaled smoke
- Carbon monoxide
- Tar
- Tobacco
- Secondhand smoke

The importance of the possible role of nicotine in the development of the following diseases

26. Possible role of nicotine in the development of smoking-related diseases:
- Extremely important

- Very important
- Important
- Not important
- No contribution

27. Possible role of nicotine in the development of lung cancer
- Extremely important
- Very important
- Important
- Not important
- No contribution

28. Possible role of nicotine in the development of tumors in other organs
- extremely important
-very important
-Important
-Not important
- No contribution
29. The possible role of nicotine in the development of Chronic Obstructive Pulmonary Disease (COPD):
- extremely important
-very important
-Important
-Not important
- No contribution

Alternative products to traditional cigarettes, without combustion

30. Have you ever heard of products without combustion and alternatives to traditional cigarettes?
- yes
-no

31. Are you aware of e-cigarettes?
-yes
-no

32. Are you aware of the presence of heated tobacco products?
-Yes
-no

33. How did you learn about alternative products to traditional cigarettes without combustion (multiple possible answers)?
-Social networks/internet
-Friends
-Relatives
-Scientific conference/dedicated workshop
- Newspaper article
34. Your opinion on these products is:
- Totally negative
- Possible
- Favorable

- I don't have enough information

35. What do you think about the training of Dental Hygienists on smoking-related issues and alternative products?
- Fundamental
- Useful
- Useless
- I don't have an opinion on the matter

Survey B

TRACKLING CIGARETTE SMOKING: LEVEL OF AWARENESS AND BEHAVIOR OF STUDENTS IN HEALTH PROFESSIONS: UNIVERSITY OF MODENA AND REGGIO EMILIA

Participant Characteristics
1. Age
2.Gender
3.Health profession:
-Healthcare Assistance
-Dietetics
-Physiotherapy
-Nursing
-Speech Therapy
-Midwifery
-Psychiatric Rehabilitation Technician
-Cardiovascular Physiopathology and Perfusion Techniques
-Biomedical Laboratory Technician
-Medical Radiology Technician for Imaging and Radiotherapy
-Occupational Therapy
-Medicine and Surgery
-Dentistry and Dental Prosthetics
-Psychology
3. Year of study in Univesity of Modena and Reggio Emilia:
-1st
-2nd
-3rd
-4th
-5th
-6th

4. Indicate the region in which you study or have studied
5. Indicate the region in which you practice the profession
6. Have you ever had specific training in smoking? - Smoking-related diseases Methods for quitting smoking - alternative products?
7. Are you a smoker?
8. If you smoke regularly, how long have you smoked? Express in months/number of years
- No, I have never smoked
- Yes, regularly
- Yes, occasionally
- No, I am an ex-smoker
9. What do you smoke?
- Traditional cigarettes
- Cigars
- Cigarillo
- Pipe
- Pre-filled e-cigarette sticks
- Refillable e-cigarette with liquids
- Heated tobacco
- Rolled or fine cut

10. How often?
-1/3 per dayszd
- 4/9 per day
- 10/15 per day
-16/ 20 per day
- >20 per day
11.If you smoke occasionally, how long have you smoked? Express in months or years
12. If you are an occasional smoker, what do you smoke?
- Traditional cigarettes
-Cigars
- Cigarillo
- Pipe
- Electronic cigarette pre-filled sticks
- Electronic cigarette with refillable liquids- Heated tobacco- Rolled or fine cut
-1/3 per day, - 4/9 per day, - 10/15 per day-16/ 20 per day- >20 per day

13. How often? Indicate the number of days/month (if you smoke >20 days per month you are not an occasional smoker)
- Traditional cigarettes
- Cigars
- Cigarillo
- Pipe
- Electronic cigarette pre-filled sticks
- Electronic cigarette with refillable liquids
- Heated tobacco
- Rolled or fine cut
-1/3 per day, - 4/9 per day, - 10/15 per day-16/ 20 per day- >20 per day
14. I am an ex-smoker. How long? Express in months-years
15.What did you smoke?
- Traditional cigarettes
- Cigars
- Cigarillo
- Pipe
- Electronic cigarette pre-filled sticks
- Electronic cigarette with refillable liquids
- Heated tobacco
- Rolled or fine cut
16. How often?
-1/3 per day
- 4/9 per day
- 10/15 per day
-16/ 20 per day
- >20 per day
17. If you are a smoker, how would you quit smoking (multiple answers possible)?
- Anti-smoking Center
- Spontaneously
- Nicotine Replacement Therapy (Nicotine gum, patches, spray)
- Pharmacologically (bupropion, varenicline, cytisine)
- Anti-smoking counseling
- Alternative therapies
- Other

Approach to smoking/use of alternative products

18. If you were a smoker of combustible products, what was the main motivation that led you to start?
- Emulation
-Personal pleasure of tobacco
-Sense of integration/belonging to a group
-Personal discomfort
-Reduction of stress
-Increased ability to concentrate
-Other
19. If you are a user of alternative products without combustion (electronic cigarette and heated tobacco), what was the main motivation that led you to start/switch to the latter (multiple answers possible)?
- Emulation
-Personal pleasure of tobacco
-Sense of integration/belonging to a group
-Personal discomfort
-Insecurity
-Reduction of stress
-Increased ability to concentrate
- Perception of lower health risk compared to traditional cigarettes
-Absence of health dangers
-Possibility of using them indoors
-Other

20. If you have switched from traditional cigarettes to smokeless products:

How long ago? Express in months or years. (e.g. 1 year)

21. Since the switch to today have you noticed any changes in your health/aesthetics?
- yes
-no

22. If yes, please tick one or more of the following boxes:
- Less/disappearance of cough in the morning
- Less/disappearance of mucus in the upper airways
- Greater ability to exercise
- Improved breath
- Improved sense of taste
- Less yellow/discolored teeth
- Smoother or firmer facial skin
- Other
23. If you use e-cigarettes, what type?
- open system (refillable tank)
- closed system (pre-filled cartridges)
- disposable, non-refillable

Knowledge

Using a scale from 1 (low risk) to 10 (high risk) and based on the information you have, indicate what, in your opinion, is the score associated with the health risk of the following products and components for smokers:

24. Health risk score for:
- Traditional cigarettes
- Electronic cigarettes (e-cigs)
-Nicotine replacement therapy
-Heated tobacco products
25. Health risk scores for smoking components
- Nicotine
- Inhaled smoke
- Carbon monoxide
- Tar
- Tobacco
- Secondhand smoke

The importance of the possible role of nicotine in the development of the following diseases

26. Possible role of nicotine in the development of smoking-related diseases:
- Extremely important
- Very important
- Important
- Not important
- No contribution

27. Possible role of nicotine in the development of lung cancer
- Extremely important
- Very important
- Important
- Not important
- No contribution

28. Possible role of nicotine in the development of tumors in other organs
- extremely important
-very important
-Important
-Not important
- No contribution
29. The possible role of nicotine in the development of Chronic Obstructive Pulmonary Disease (COPD):
- extremely important
-very important
-Important
-Not important
- No contribution

Alternative products to traditional cigarettes, without combustion

30. Have you ever heard of products without combustion and alternatives to traditional cigarettes?
- yes

-no

31. Are you aware of e-cigarettes?
-yes
-no

32. Are you aware of the presence of heated tobacco products?
-Yes
-no

33. How did you learn about alternative products to traditional cigarettes without combustion (multiple possible answers)?
-Social networks/internet
-Friends
-Relatives
-Scientific conference/dedicated workshop
- Newspaper article
34. Your opinion on these products is:
- Totally negative
- Possible
- Favorable
- I don't have enough information

35. What do you think about the training of Dental Hygienists on smoking-related issues and alternative products?
- Fundamental
- Useful
- Useless
- I don't have an opinion on the matter
